# Reliability of cortical lesion detection on double inversion recovery MRI applying the MAGNIMS-Criteria in multiple sclerosis patients within a 16-months period

**DOI:** 10.1371/journal.pone.0172923

**Published:** 2017-02-24

**Authors:** Tobias Djamsched Faizy, Christian Thaler, Tim Ceyrowski, Gabriel Broocks, Natascha Treffler, Jan Sedlacik, Klarissa Stürner, Jan-Patrick Stellmann, Christoph Heesen, Jens Fiehler, Susanne Siemonsen

**Affiliations:** 1 Department of Diagnostic and Interventional Neuroradiology, University Medical Center Hamburg-Eppendorf, Hamburg, Germany; 2 Department of Neurology, University Medical Center Hamburg-Eppendorf, Hamburg, Germany; 3 Institute for Neuroimmunology and Clinical MS Research, University Medical Center Hamburg-Eppendorf, Hamburg, Germany; Universitat Ulm, GERMANY

## Abstract

**Purpose:**

In patients with multiple sclerosis (MS), Double Inversion Recovery (DIR) magnetic resonance imaging (MRI) can be used to identify cortical lesions (CL). We sought to evaluate the reliability of CL detection on DIR longitudinally at multiple subsequent time-points applying the MAGNIMs scoring criteria for CLs.

**Methods:**

26 MS patients received a 3T-MRI (Siemens, Skyra) with DIR at 12 time-points (TP) within a 16 months period. Scans were assessed in random order by two different raters. Both raters separately marked all CLs on each scan and total lesion numbers were obtained for each scan-TP and patient. After a retrospective re-evaluation, the number of consensus CLs (conL) was defined as the total number of CLs, which both raters finally agreed on. CLs volumes, relative signal intensities and CLs localizations were determined. Both ratings (conL vs. non-consensus scoring) were compared for further analysis.

**Results:**

A total number of n = 334 CLs were identified by both raters in 26 MS patients with a first agreement of both raters on 160 out of 334 of the CLs found (κ = 0.48). After the retrospective re-evaluation, consensus agreement increased to 233 out of 334 CL (κ = 0.69). 93.8% of conL were visible in at least 2 consecutive TP. 74.7% of the conL were visible in all 12 consecutive TP. ConL had greater mean lesion volumes and higher mean signal intensities compared to lesions that were only detected by one of the raters (p<0.05). A higher number of CLs in the frontal, parietal, temporal and occipital lobe were identified by both raters than the number of those only identified by one of the raters (p<0.05).

**Conclusions:**

After a first assessment, slightly less than a half of the CL were considered as reliably detectable on longitudinal DIR images. A retrospective re-evaluation notably increased the consensus agreement. However, this finding is narrowed, considering the fact that retrospective evaluation steps might not be practicable in clinical routine. Lesions that were not reliably identifiable by both raters seem to be characterized by lower signal intensity and smaller size, or located in distinct anatomical brain regions.

## Introduction

Multiple sclerosis (MS) is a chronic-demyelinating inflammatory disease, which is not only associated with an extensive load of white matter lesions (WML) but also with a considerable grey matter involvement [1–3]. In the last couple of years, the investigation and the assessment of grey matter (GM) lesions in multiple sclerosis patients (particularly cortical lesions (CL)) has become subject of extensive research. Recent studies have claimed, that the progression of the patients´ disease course, the degree of physical disability and the extend of cognitive impairment are closely associated with the degree of grey matter damage [4–10].

The *in vivo* assessment of CLs has substantially been improved i.a. by advanced magnetic resonance imaging (MRI) techniques like Double inversion recovery (DIR), which provides high-contrast depictions of MS lesions and cerebral grey matter [11]. As an add-on to standard imaging, DIR may improve the visibility of grey matter and especially CL in comparison to standard MRI techniques [12]. However, even with the additional utilization of DIR sequences, the differentiation between distinct cortical lesion types, such as pure intracortical lesions, leucocortical lesions and juxtacortical lesions was found to be challenging [13, 14]. Furthermore, when evaluated by two or more investigators, considerable differences of cortical lesion detection rates have been reported and reproducibility of CL was found to be poor in consecutive MRI examinations [15]. Also, only a minority of histopathologically verified CLs were found to be actually detectable on DIR [16]. This points out the necessity to critically discuss the reliability of CLs detectability on DIR. In 2011, the MAGNIMS expert group has established consensus guidelines for the assessment of MS lesions (including cortical grey matter lesions) in *in vivo* MRI examinations. These guidelines shall contribute to improve the sensitivity when scoring cortical lesions in MS patients and aim to standardize diagnostic approaches [15, 17, 18]. Recently, in January 2016, an updated version of the MAGNIMS consensus MRI guidelines for the diagnosis of multiple sclerosis has been published [16], which further underlines the significance and importance of a proper assessment of CLs and its potential role for the individual patient.

The aim of this study was to evaluate the reliability of cortical lesion detection on DIR in a 16-month prospective frequent MRI study using the MAGNIMS scoring criteria for CLs. We aimed to investigate the visibility of CLs at 12 consecutive MRI examinations and tested, how reliable CL can be identified by two different raters in a longitudinal study setup. Furthermore, we evaluated the impact of lesions´ volumes and lesions´ relative signal intensities on their detectability. We hypothesized that the reliability of cortical lesion identification is dependent on lesion size and signal intensity and that anatomical lesion localizations influence the visibility and therefore the detectability of CLs.

## Methods

### Patients´ characteristics

Twenty-six patients, all with Relapsing-remitting Multiple Sclerosis (RRMS) diagnosed with definite MS based on the 2010 revisions of the McDonald criteria [19] were included in our study as part of a phase IIa clinical trial on boswellic acids to treat active relapsing-remitting MS in a baseline to treatment design (clinical trial number: NCT01450124). The study was approved by the local research Ethical Committee Hamburg (Ethik-Komission der Ärztekammer Hamburg) and the BfArm (federal authority), following the guidelines of the Declaration of Helsinki and written informed consent was given from every subject.

MRI examinations were performed at 12 different time-points over a 16-month period and scans were conducted at months 1–7, 9–12 and 16. Baseline MRIs were conducted at “month 1”, treatment onset was at “month 5”. Patients´ demographic data are displayed in Table 1.

**Table 1:** Demographic data of the study cohort.

Subject demographics	Mean value ± sd	Range
**Age**	38.31 ± 10.81	23–56
**EDSS**	1.9 ± 0.7	1–3.5
**Disease duration (in years)**	6.23 ± 7.35	0.8–26
**Gender (male / female)**	4 / 22	n.a.
**Number of cortical lesions**	9.86 ± 6	1–37
**Lesion volume [mm**^**3**^**]**	29.04 ± 39.06	3.67–531.8
**Relative signal intensity**	78.5 ± 13.48	4.91–140.18

Table displays the mean values of patients´ demographic data distributed over all 12 time-points. EDSS = Expanded Disability Status Scale. Mean number of cortical lesions and lesion volumes include both lesion types: conL and non-conL.

### MR image data acquisition

MRI scans were performed on a 3T MR Scanner (Skyra, Siemens Medical Systems, Erlangen, Germany) using a 32 channel head and neck coil. Our standard clinical routine MS protocol consisted of axial 2D T2w turbo spin echo (TSE) images acquired with TR = 2800ms, TE = 90ms, 43 slices, Matrix: 192x256, slice thickness = 3mm and in plane resolution = 0.5x0.5mm2, turbo factor = 5. 3D-FLAIR images with TR = 4700ms, TI = 1800ms, TE = 390ms, 192 slices, slice thickness = 1mm, matrix = 256x256. T1-MPRAGE pre and post Gadolinium with TR = 1900ms, TE = 2.43ms, TI = 900ms, slice thickness = 1mm, matrix = 256x256x192, voxel size = 1x1x1mm and Gd-dose of 0.2ml per kilogram of body weight. In addition to our standard clinical protocol. 3D DIR sequences (pre Gadolinium) were acquired with TR = 7500, TE = 321.0, TI = 3000/450, 192 slices, slice thickness = 1.4mm, matrix = 256x256, in plane resolution = 1.4x1.4 mm^2^.

### Data processing and image analysis

#### Cortical lesion identification

The acquired image data, along with the DIR sequences, were processed using the software package fsl 5.0 (Analysis Group, FMRIB, Oxford, UK). Brain extracted DIR images were intensity normalized to a common mean and image data were set to equal intensity levels in order to minimize measurement deviations on DIR data and to assimilate the acquired image data. Two experienced neuroradiologists (T.F. and T.C.) read the images applying the MAGNIMS criteria for cortical lesion scoring [15]. Testing of intraobserver variability was performed similar to the approach in [20] calculating a coefficient of variances for every rater. Mean intraobserver variabilities for rater 1 and 2 were 1.6% and 1.8% respectively. In a first reading step (first reading), the raters were blinded to all examination time-points and MRI scans were evaluated in random order by each rater separately. CLs were marked on each scan using the software Analyze 11.0 (Analyze Direct, Overland Park, KS, USA) by each of the raters and total lesion numbers were determined for each scan-time-point and patient. Subsequently, every single CL was followed through all scan time-points (with a side-by-side comparison of all consecutive time-points), in order to assign them to an identical corresponding lesion in any other MRI scan of the same patient. CL that were similarly visible in two or more MRI scans of the same patient were counted as one CL, either detected by rater 1 or 2. A side-by-side evaluation was also used to identify CL that were detected by both raters at any of the time-points (first agreement). In a second step, those CLs that were identified by only one of the raters were then presented to the rater that did not identify this particular lesion in the first reading and the scoring decision was re-evaluated. Subsequently, the readers had the opportunity to retrospectively confirm the lesion and therewith add it to the count of consensus lesions (conL). After the second reading, all CLs that still were detected by only one of the two readers were defined as “non-consensus lesions (non-conL)”. Subsequently, the frequency of the conLs´ visibility for each scan time-point was evaluated, i.e., the number of time-points each lesion was visible in was documented for each patient.

#### Lesion nomenclature

According to the 2016 revised MAGNIMs criteria [16], intracortical lesions (ICL) (with no visual affection of the juxtacortical or subcortical cerebral white matter (WM)), leucocortical lesions (LCL) (which similarly affected both, the cerebral cortex and the adjacent white matter) and juxtacortical lesions (JCL) were all merged into one lesion class (in the MAGNIMS paper [16] referred to as “cortical/juxtacortical lesions”).

#### Lesion characterization

Each cortical lesion was manually defined by both raters independently as a region of interest (ROI) using a seed based region growing algorithm (Analyze 11.0, Analyze Direct, Overland Park, KS, USA). Mean DIR signal intensity was documented for each lesion at every time-point it was visible. Furthermore, four ROIs were placed in the frontal and occipital cortices on both hemispheres in each DIR scan of each patient and time-point (i.e. in regions, which evidently showed no CLs) to obtain intensity values for normal appearing cortical regions. For each cortical lesion, a relative signal intensity ratio (intensR) was calculated by dividing each cortical lesion´s mean intensity value by the corresponding mean value derived from the normal appearing cortical regions.

Furthermore, the anatomic location of CLs was documented. Lesions were categorized into the following regions: frontal, parietal, insular lobe, occipital, temporal and infratentorial.

**Fig 1:**
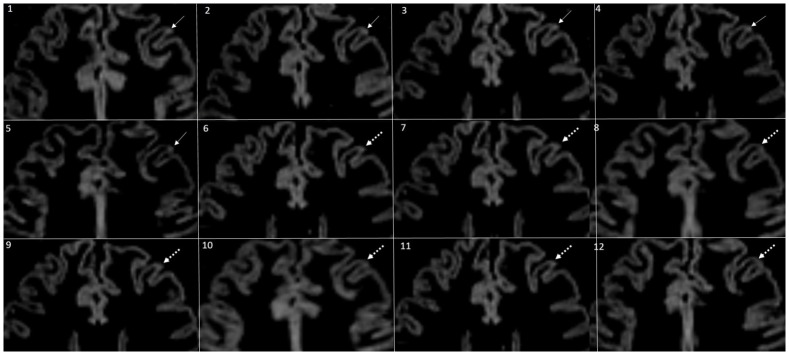
Visibility of a CL over all 12 time-points. Fig 1 depicts a relatively small cortical lesion followed over all 12 time-points. The CL was scored by consensus (conL) in the first 6 TP (white arrow). From TP 7 to TP 12 (dotted white arrows), the specific CL was no longer detected by both of the raters consensually (non-consenus lesion).

**Fig 2:**
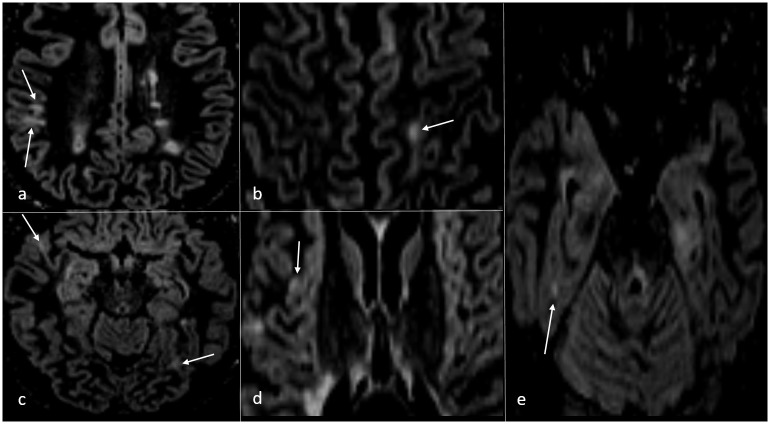
Overview of different CL types in distinct brain anatomical brain regions. Fig 2 displays different cortical lesion types in various anatomical brain regions marked by white arrows. Lesions appeared with varying volumes and signal intensities differed between distinct CLs.

Also, CLs volumes were determined lesion-wise and patient-wise at each TP. Figs 1 and 2 exemplify the visualization of CLs on DIR images.

### Statistical analysis

Number of lesions, mean signal intensities and estimates of lesion volumes are presented as mean ± standard deviation (sd). Student´s unpaired t-test was used for normally distributed data to compute statistical differences between groups for volume and intensity measurements. For not normally distributed data (like CL volumes and CL signal intensities), differences between two samples were computed with non-parametric Mann-Whitney U-Test. Chi-square test was applied to test for associations between consensus or non-consensus ratings and lesion localizations. To test and compare the inter-rater agreements after the first reading step and after retrospective re-evaluation of CLs, Cohen´s Kappa (by definition of Landis and Koch) [21] was computed. The intraobserver variability was calculated using the coefficient of variation similar to the approach in [20]. Univariate ANOVA test was computed to determine if mean lesion volumes or mean relative lesion signal intensities differed among the different anatomical brain regions. For statistical significance, an alpha error of p < 0.05 was assumed.

## Results

### Number of cortical lesions detected by the readers

A total number of 3076 random CL were identified by either the first or second rater in all 26 MS patients among all 12 TP. The mean number of CL detected by both raters per patient in all 12 longitudinal MRI examinations was 9.86 ± 6 lesions (conL and non-conL) with a median of 8.5. The range of CL identified per patient was 1–37. After the CL were assigned longitudinally in a side-by-side comparison, 334 different CL were identified, detected by either rater 1 or 2. Table 2 provides an overview of the numbers of CLs that were identified per rater and reading step. During the first reading, 230 CLs were identified by rater 1 and 251 CL were identified by rater 2 in all 26 patients throughout all 12 MRI examinations. After the first reading, 160 out of 334 CL were identified by both raters. Kappa score for inter-rater agreement after the first reading was κ = 0.48 ± 0.01 (moderate agreement). During the second re-evaluation step, an additional number of 35 CL were retrospectively verified by rater 1, and another 38 CL were retrospectively verified as lesions by rater 2. Thus, the total number of CL detected by rater 1 increased to 265 CL and 289 CL for rater 2 respectively. After this re-evaluation step, the corresponding number of consensus CL increased to 233 CL. The corresponding kappa score was κ = 0.69 ± 0.12 (substantial agreement). Accordingly, 101 of the 334 CLs were categorized as non-consensus lesions (non-conL).

**Table 2:** Number of cortical lesions identified after the first reading and re-evaluation.

Number of CL	Rater 1	Rater 2	Consensus lesions (conL)
Number of CL detected in the first reading	230	251	160 (48%)
Number of retrospectively agreed CL	35	38	n.a.
Number of CL detected after re-evaluation	265	289	233 (69%)

Table 2 displays the number of cortical lesions (CL) identified by rater 1 or rater 2 in the first reading step and after the retrospective re-evaluation of lesions that were only detected by one of the raters in the first reading. Consensus lesions were defined as lesions identified by both raters.

### Detectability of CLs in relation to lesion volumes and signal intensities

**Fig 3:**
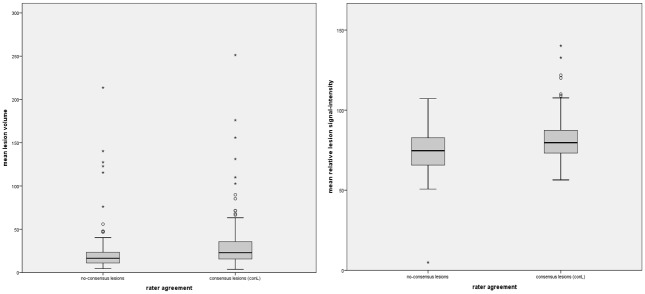
Differences of mean cortical lesion volumes and mean signal intensities between conL and non-consensus lesions. Boxplots in fig 3 display the mean lesion volume (in mm^3^) and mean signal intensities of CLs that were detected by only one of the two raters (non-conL; left side) vs. CLs that were detected by both raters (conL; right side). Mean volumes and mean relative signal intensities of conL were significantly higher (p<0.05) compared to non-conL.

There was a significant difference of mean cortical lesion volumes in conL compared to non-conL. Mean conL volume (32.50mm^3^ ± 44.33mm^3^) was significantly higher in comparison to non-conL volume (23.20mm^3^ ± 28.28mm^3^, p = 0.037). Also, conL showed a significantly higher mean relative signal intensity (81.1 ± 12.40) than non-consensus lesions (71.10 ± 14.14, p<0.001). Fig 3 displays the relative signal intensity and volume of conL in comparison to non-CL.

### Longitudinal visibility of consensus lesions

74.7% of all 233 conL were visible and thus detected by definition by both raters at all 12 consecutive time-points. 2.6% and 4.3% of the conL were visible in 11 and 10 consecutive TP, respectively. Only 16% of the conL were apparent in less than 6 consecutive TP. Table 3 summarizes the frequencies of conL detectability over the time course.

**Table 3:** Frequency of consensus lesions´ detection over the time-course.

Consecutive time-points	Number of conL	Percentage
**12**	174	74.7%
**11**	6	2.6%
**10**	10	4.3%
**9**	3	1.3%
**8**	3	1.3%
**7**	0	0%
**6**	1	0.4%
**5**	0	0%
**4**	15	6.4%
**3**	3	1.3%
**2**	4	1.7%
**1**	14	6%
**Total**	233	100%

Index: Consecutive time-points = number of immediate consecutive time-points in which consensus lesions (conL) were visible; Middle and right column are displaying the absolute frequency and the percentage of conL visible at the specific time-points.

### Lesion detectability in association to brain regions

Sixty-six of the conL were identified in the frontal lobe and 53 conL were located in the parietal lobe. Six conL were located in the insular and 27 conL were found in the occipital lobe. Sixty conL were identified in the temporal lobe and 15 lesions were identified in the infratentorial brain region. In contrast, 21 non-conL were located in the frontal lobe, 24 CLs in the parietal lobe, 9 lesions in the insular, as well as 6 CLs in the occipital lobe, 23 CLs in the temporal lobe and 24 CLs in the infratentorial brain region.

Compared to non-conL, the number of conL identified in the frontal, parietal, occipital and temporal lobes was significantly (p<0.05) higher. There was no significant difference between conL and non-conL in the frequency of lesions detected in the insular (p = 0.8) and infratentorial brain region (p = 0.98) compared to non-conL.

### Lesion intensities and volumes in different brain localizations

ConLs´ mean relative signal intensities in the insular lobe were significantly higher compared to the mean relative signal intensities of conL in the frontal (p = 0.02), parietal (p = 0.008) and temporal lobes (p = 0.045) but did not differ significantly from conL in the occipital (p = 1.00) and infratentorial brain regions (p = 0.99). Mean conL volumes did not show significant differences among all lesion localizations (p>0.05 for all localizations).

## Discussion

In our study, we investigated the reliability of CL detection on a 3 Tesla Double Inversion Recovery sequence in a longitudinal survey with frequent short-term follow-up examinations applying the MAGNIMs consensus criteria for CL scoring [15]. CLs have gained interest in MS research, but a reliable identification of CLs remains a matter of discussion. Here, we primarily observed only a moderate rater agreement between two experienced readers. However, this shortcoming might be reduced by implementing a second consensus evaluation step as we did in this study. Also, the interrater-agreement seemed to increase with greater lesion volumes and signal intensity.

The identification of CLs on DIR scans can be challenging, due to their specific anatomical location in the cerebral cortex [13, 14] and remarkable differences of lesion volumes and distinct lesion subtypes [1, 15, 22]. Furthermore, the detection of CLs can be influenced by a various number of technical and methodological preconditions, such as the underlying field strength of the MRI scanner, sequence parameters, the utilized scan protocol and especially by the applied scoring criteria for CL identification [15–18]. However, there are no studies focusing on the evaluation of consistency and reliability of CL identification over a longitudinal time course taking into account different conditions such as volume and intensity or location that might positively or negatively influence the detectability of CLs in general.

In the first evaluation step of our study only a moderate (48%) inter-rater agreement was achieved. Retrospective evaluation increased the inter-rater agreement to 68%. This re-evaluation was performed in order to minimize the amount of CLs that were simply missed during the first reading. This step provided important information about the reliability of CL detection in general, since–at least in this study- we implied that only CL that have been identified by both raters consistently could be considered as “real/reliable”. Thus, it is noteworthy that a retrospective evaluation of particular CLs seemed to increase the accuracy of lesion detection notably. On the other hand, the implementation of a second re-evaluation step must be discussed critically, because it might not be applicable in clinical routine, since it is highly time consuming and expensive. Furthermore, it seems that under conditions comparable to clinical routine (only one evaluation step) CL detection on DIR still seems to be challenging, since only a moderate inter-rater agreement was achieved. Poor inter-rater agreement was reported in a previous study when scoring CLs on DIR [15]. However, multiple evaluation steps of CLs on DIR in order to minimize intra-rater or inter-rater deviations seem to be common [20, 23]. Referring to the first consensus recommendations for MS CL-scoring from 2011 [15], complete rater-agreement was only reached in 19.4% of the assessed lesions. More than half of the readers agreed on slightly more than half of the CL scored (54%) [15]. However, in the referred study of Guerts et al. [15], CL ratings was performed in 5 groups of 2–3 readers each on the basis of a heterogeneous dataset of DIR images, as part of a multicenter study. In contrast, in our study, all patients were examined on the same 3T scanner without performing any major upgrade during the course of the study. Heterogeneity of data acquisition and multicenter approaches might explain the differences to previous publications.

Our results indicate that lesions, which were consistently identified by both raters, were larger and showed a higher mean relative signal intensity. An explanation for this finding could be the intrinsic poor signal-to-noise ratio, a known problem of DIR sequences, which might aggravate the detection of small or more hypointense lesions [13]. Furthermore, CL detectability is known to be somehow restricted in brain regions, where “artificial” hyperintensities are most likely to occur on DIR, for example in the insular, temporal or occipital lobes [24]. We assume that larger or brighter lesions might be less prone to those artefacts own to their prominent visual appearance. In line with recent studies, we also detected a similar topographic distribution of CLs throughout the brain [24]. In our study, a higher number of CLs was detected by consensus (conL) in the frontal and temporal lobe as well as in the parietal and occipital lobes compared to the corresponding number of non-conL. In the insular and infratentorial brain regions, no significant difference was noted in the number conL and non-conL. This might suggest a more reliable detection of CL in most of the neocortex areas. In contrast, a reliable identification of CLs seems to be challenging in brain regions where CLs occur less frequent. Furthermore, it appears that CLs, which are located in areas that are technically prone to artefacts—like the insular ribbon—are less reliably detectable by different raters. However, the distributions of the identified conL are comparable to former investigations of RRMS patients [24, 25]. Mean CL volumes and signal intensity of the consensually detected lesions did not differ significantly between the anatomical brain regions. Therefor CL´s volume and intensity on their own cannot be the only parameters accounting for the detectability of CLs. Presumably the synergy between multiple parameters including volume, intensity and anatomic brain region taken together significantly influence CL detectability.

Studies on longitudinal detectability of CLs are rare. However, recent studies sought to investigate the evolution of CLs and their influence on the patient´s clinical course in longitudinal surveys.

In 2009, Calabrese et al. [23] investigated cortical lesions in a homogeneous study cohort of fourty-eight patients with primary progressive multiple sclerosis (PPMS) in a two-year longitudinal study. At baseline, CLs were found in 81.2% of the patients. Another three-year longitudinal study, also by Calabrese et al. [20] investigated the evolution of CLs in a study cohort of RRMS and secondary progressive multiple sclerosis (SPMS) patients. At baseline, CLs were detected in 64.4% of RRMS and 74.2% of SPMS patients respectively. Though the main focus of these studies was not on the detectability of CLs in general, the authors reported that the majority of the CLs that were identified at baseline were also detectable on follow-up examinations. In our investigation, all of the 26 RRMS patients showed at least one conL at baseline scan and the majority of the conL (74%) was identified on all 12 MRI scans. In addition, lesions that were detected with a frequency of 1–11 in consecutive TP during the follow-up period were considered as new lesions, which predominantly also seem to be reliably detectable on DIR scans. However a considerable number of CL has been detected by consensus in 9, 10 or 11 consecutive TP. An explanation for that may be that one of the readers might have disagreed on a CL that was presented to him in the re-evaluation step. This might explain, why some of the CL appear to have a “gap” at certain TP and were not detected by both of the raters in all 12 consecutive TP. Regarding the fact that some con-CL were detected in 6 or less consecutive TP, we assume that these CL might have emerged newly among the time course. Moreover, the mean amount of new CL per patient among the follow-up is relatively comparable to related studies, which might support our assumption [20, 23, 26]. Nevertheless, there is still a considerable number of CLs (n = 101) that was not detected by both of the readers but albeit, by one of the readers. An explanation for that might be that besides the fact that the process of CL detection itself has improved remarkably in the last couple of years (especially due to the implementation of improved scoring recommendations), some methodological and procedural elements will remain a matter of interindividual interpretation. As mentioned above, the identification of CLs on DIR is still very challenging due to known problems such as the intrinsic low signal-to-noise-ratio, common pulsation artefacts and focal artefacts in certain brain areas. It seems for now, that some scoring decisions will remain a matter of individual assessment, even when a scoring guideline is followed. That may also explain why even some considerably large lesions (lesion volume>100mm^3^) were not detected by both raters consensually. This circumstance might lower the clinical applicability of DIR sequences for a reliable identification of CLs, since multiple rating steps (like a retrospective re-evaluation of CLs) to reach higher levels of agreements might not be suitable for the clinical routine. A continuing evaluation and further improvements of both, technical conditions and scoring recommendations might reduce the remaining issues for a reliable identification of CLs on DIR images.

Our study also holds some limitations: First, although the utilization of DIR sequences has significantly increased the detection rate of CLs in MRI examinations, only a minority (~20%) of histologically verified lesions (especially subpial lesions, which appear to be the most common CLs in MS-patients) can be detected on *in vivo* MRI scans [13, 22, 27, 28]. Our study lacks of a comparable gold standard and/or histopathological correlations, so any claims about the validity of the detected CL, especially during the second re-evaluation step, need to be mitigated. Also, the application of multimodal MRI sequences at once seems to be at least equal to the use of DIR in the detection of CLs [29]. Second, the comparability to other recent DIR studies is still poor, since the MRI scan parameters and image characteristics are not well harmonized across different research centers and between different manufacturers [16]. The possible influence of newly appearing or disappearing CLs during the time course on the patients´ disease process needs further investigations. Further, extended studies with correlations to clinical parameters will be needed.

## Conclusion

The application of DIR sequences seems to be a valid method for a reliable detection of CLs in MS patients, but also holds some evident limitations. Stepwise evaluations with multiple raters and re-evaluation steps increase reliability but might only be applicable in study settings and not in clinical routine. The assessment of CL on DIR under conditions comparable to clinical routine still lacks accuracy revealing only moderate inter-rater agreements. CLs with higher mean lesion volumes and higher relative signal intensities are more frequently identified by consensus. Additional improvements of technical and methodological processes might further improve the feasibility and reliability of DIR for CL detection.

## Supporting information

S1 File Minimal data set. TP = time-point (1–12); Localisation = (1 = frontal lobe; 2 = parietal lobe; 3 = insular; 4 = occipital lobe; 5 = temporal lobe).
(XLS)
